# Predicting phonological information in language comprehension: evidence from ERP representational similarity analysis and Chinese idioms

**DOI:** 10.1093/cercor/bhad209

**Published:** 2023-06-14

**Authors:** Wei Wei, Zirui Huang, Chen Feng, Qingqing Qu

**Affiliations:** Key Laboratory of Behavioral Science, Institute of Psychology, Chinese Academy of Sciences, Beijing 100101, China; Sino-Danish College, University of Chinese Academy of Sciences, Beijing 100040, China; Key Laboratory of Behavioral Science, Institute of Psychology, Chinese Academy of Sciences, Beijing 100101, China; Key Laboratory of Behavioral Science, Institute of Psychology, Chinese Academy of Sciences, Beijing 100101, China; Department of Psychology, University of Chinese Academy of Sciences, Beijing 100040, China; Key Laboratory of Behavioral Science, Institute of Psychology, Chinese Academy of Sciences, Beijing 100101, China; Department of Psychology, University of Chinese Academy of Sciences, Beijing 100040, China

**Keywords:** Chinese idiom, ERP, phonological prediction, prediction, representational similarity analysis

## Abstract

Do comprehenders predict the meaning and even the phonological form of upcoming words during language comprehension? With a growing body of evidence suggesting that semantic representations may be predicted, the evidence for phonological prediction is less clear and largely derived from studies conducted in languages utilizing an alphabetic script. In this research, we aim to examine the prediction of phonological information in the processing of Chinese idioms through the use of ERP representational similarity analysis (RSA). The study utilizes four-character Chinese idioms, and phonological overlap was manipulated by varying the syllable at the idiom-final part between idiom pairs so that pairs of idioms share a syllable (i.e. within-pairs) or not (between-pairs). We quantified the similarity between patterns of neural activity of idioms for within- and between-pairs. RSA results revealed greater similarity in neural activity patterns for idioms within-pairs, compared with between-pairs, and critically this similarity effect was observed prior to the presentation of the phonological similarity, providing evidence for the pre-activation of upcoming phonological information, under circumstances that encourage predictive processing.

It has been posited that prediction plays a crucial role in language comprehension and is a fundamental computational principle (Kuperberg and Jaeger 2016). This notion assumes that individuals generate predictions at various levels of representation during language comprehension, including high-level grammatical or semantic representations that are critical to the ultimate goal of language comprehension, as well as fine-grained phonological representations. Despite a growing body of research that has demonstrated the existence of prediction in meaning or syntax during language comprehension (Altmann and Kamide 1999; DeLong et al. 2005; Staub and Clifton 2006; Laszlo and Federmeier 2009; Ito et al. 2016a), the evidence for phonological prediction is less clear and often inconsistent (DeLong et al. 2005; Ito and Husband 2017; Ito et al. 2018; Nieuwland et al. 2018; for a comprehensive review, see Nieuwland 2019; Li et al. 2022). In this study, we aimed to provide neural evidence for the prediction of phonological information in language comprehension by using electroencephalography (EEG) in combination with representational similarity analysis (RSA).

The pre-activation of word form has been demonstrated through the eye-tracking visual world paradigm, which involves presenting spoken sentences and visual scenes, and analyzing the predictive eye movements prior to hearing the target words. In Ito et al. (2018), participants listened to sentences containing highly predictable words (e.g. “cloud” in “The tourists expected rain when the sun went behind the...”) and viewed a scene that contained a target object corresponding to the predictable word, a phonological competitor object that was phonologically related to the predictable word, or an unrelated object. Results indicated that participants made more fixations to the phonological competitor objects prior to hearing the target words, compared with the unrelated objects, suggesting that participants pre-activated the phonological information of the target words, leading to increased fixations on the phonological competitors. However, close replications of Ito et al. (2018) have produced mixed results, with some positive findings (Kukona 2020; Li et al. 2022), and some null findings (Ito and Husband 2017; Ito and Sakai 2021). As a result, the findings from the visual world paradigm present a complex picture.

The study by DeLong et al. (2005) provides a significant contribution to the empirical evidence for phonological prediction in language comprehension. The study adopted the English phonological rule that the articles “a” and “an” are used before consonant- and vowel-initial words, respectively. Participants were presented with highly predictable sentences, followed by highly predictable or less predictable articles and nouns (e.g. “The day was breezy so the boy went out to fly a/an kite” or “an airplane”). The results indicated that the N400 amplitude elicited by a given word was systematically related to cloze probability, both for nouns and, notably, for pre-noun articles. This was taken as evidence that participants pre-activated phonological information of nouns and preceding articles, with prediction error arising from falsification of the prediction. Several studies have attempted to replicate the phonological prediction effect observed in DeLong et al. For instance, Martin et al. (2013) used the a/an paradigm but conducted a different analysis, and found that the comparison of N400 for expected versus unexpected articles revealed a larger N400 for unexpected articles in L1 speakers, but not in L2 speakers (see DeLong et al. 2012 for the failure of replicating the pre-noun article effect in older adults). A recent large-scale direct replication study (*n* = 334 from nine laboratories), Nieuwland et al. (2018) failed to replicate the graded phonological prediction effect for pre-noun articles. This result pattern was observed in both a direct replication analysis that duplicated the original study’s analysis and a pre-registered single-trial analysis that modeled variance at the item and subject level. However, it should be noted that the absence of evidence for phonological prediction from the a/an paradigm does not necessarily exclude the presence of such prediction during language comprehension. The failure to detect the effect of phonological prediction could stem from the limitations of the a/an paradigm, as articles are not always immediately followed by a noun and are often directly followed by pre-nominal adjectives. In later cases, articles are only diagnostic about the following adjective, rather than the noun itself. This could result in less-expected articles not generating confirmation of prediction, leading to a lack of processing difficulty and the graded N400 effect. Therefore, there is currently no consistent evidence from the a/an paradigm regarding the presence of phonological prediction in language comprehension.

The word-form prediction is also supported by the N400 form-related effect. In a study by Ito et al. (2016b), participants read highly predictable sentences, such as “The student is going to the library to borrow a...”, followed by either a predictable target word (e.g. book), an unexpected word that was related to the predictable word in form (e.g. hook), an unexpected word that was semantically related to the predictable word (e.g. page), or an unrelated word (e.g. sofa). The results showed that both semantically related words and word-form related words elicited a smaller N400 compared with unrelated words, and the N400 effect depends on cloze probability. This reduction in N400 effect has been interpreted as pre-activation of meaning or form of target words, thereby making the processing of meaning- or form-related words easier. But it has been debated whether the reduced N400 truly reflects prediction or is simply a result of the greater plausibility and easier integration of target words with prior context. Recently, some researchers have challenged the view that the predictability-dependent N400 purely reflects the effects of prediction, and instead suggest that the N400 effect can be explained by integration processes. A large-scale study that used a mixed-effect multiple regression analysis found dissociable effects of prediction and integration on the N400 (Nieuwland et al. 2020). In addition to the N400, several ERP and MEG studies have reported early pre-N400 brain responses (M100, P130, N1, P2, N200/PMN, N250, P300) as evidence of word form prediction (e.g. Dikker et al. 2009, 2010; Vespignani et al. 2010; Gagnepain et al. 2012; Kim and Lai 2012; Sohoglu et al. 2012; Dikker and Pylkkänen 2013). It should be noted that these early effects have been critically reviewed by Nieuwland (2019).

Recently, the presence of prediction in the brain has been indicated by pre-stimulus brain responses. Several studies have reported a negative potential shift, beginning hundreds of milliseconds prior to the presentation of predictable stimuli, which is considered as the brain’s direct evidence of prediction (Grisoni et al. 2016, 2017, 2019; León-Cabrera et al. 2017, 2019; see Pulvermüller and Grisoni 2020 for a review). Critically, the cortical sources of the prediction potential reflect quite specific perceptual and semantic features of predictable stimuli (e.g. Grisoni et al. 2017, 2019). For instance, the specific topography of the prediction potential may index whether predictable action verbs are hand-relevant or face-relevant, suggesting that the prediction potential is related to semantic pre-activation. Of particular relevance to the present investigation is the question of how the prediction of phonological information is related to the prediction potential. A recent investigation by Grisoni and Pulvermüller (2022) has shed light on this question by showing that prediction concerning forthcoming speech sounds, which are provoked by frequently repeated spoken stimuli, can also evoke analogous ERP potentials.

Another line of research has employed RSA (Kriegeskorte et al. 2008) to investigate prestimulus predictive brain activity. RSA measures similarity in patterns of neural activity before or after encountering the target word and assumes that similarities between items elicit similarities in brain activity patterns. In a study by Wang et al. (2018), the authors used RSA and magnetoencephalography (MEG) to examine whether readers can predict specific words while reading highly constraining sentences and found that the patterns of neural activity were more similar when the same words were predicted than when different words were predicted, critically before the onset of the predicted words. Another study by Hubbard and Federmeier (2021) used EEG and RSA to compare the similarity patterns of final words in constraining sentences to those of pre-final words. The logic is that if semantic features of final words is pre-activated prior to the final word, then neural representations of the final word should already appear during the processing of the pre-final word, thus producing greater similarity between pre-final and final words. It was found that neural similarity with the final word was increased following the processing of the pre-final word, which was modulated by expectancy of final words and sentence constraint. This further supports the prediction of the final word’s semantic representation. In a recent study, Wang et al. (2020a) found evidence for the prediction of animacy features of upcoming words when sentence contexts only constrain the coarse-grained semantic animacy feature, demonstrating the prediction of coarse-grained semantic features beyond the prediction of specific words. However, it should be noted that the RSA studies mentioned above mainly focus on the prediction of semantic representations, with little research being conducted on the neural activity similarity elicited by phonological pre-activation.

In this study, we aimed to investigate phonological prediction in Chinese idioms reading using ERP and RSA. RSA presents a promising approach to investigate prestimulus predictive effects, as demonstrated in recent studies by Huang et al. (2023), Hubbard and Federmeier (2021), and Wang et al. (2020b). RSA is grounded in the assumption that similarity of neural activity evoked by the processing of stimuli can be influenced by the similarity structures of those stimuli. On the basis of this assumption, it is expected that phonologically similar stimuli would induce greater similarity in neural activity. By analyzing the similarity of neural activity prior to stimulus presentation, one can investigate whether this increased neural similarity associated with phonological similarity is evident before the presentation of the phonologically similar stimuli, thereby providing evidence for phonological prediction. Moreover, the study utilizes Chinese idiom because the canonical structure and word order of idiomatic expressions make them particularly well suited for investigating linguistic prediction. The Chinese language is rich in idioms, with over 18,000 documented in the Complete Dictionary of Chinese Idioms (Wang 2008), which are frequently used in daily life. Most Chinese idioms consist of four characters and are structurally fixed (e.g. 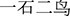,/yi1shi2er4niao3/, one rock two birds), with some also used in English, such as “kill two birds with one stone.” In our task, four-character Chinese idioms were presented, with the first two characters displayed first and the last two subsequently. The manipulation of phonological overlap was performed in the third character among idioms, with some idioms sharing the same sound at the third character (i.e. within-pairs), while others did not (between-pairs). We quantified the similarity of patterns of neural activity before and after the presentation of the third character. Phonological similarity among characters should elicit greater neural similarity, thus similarity of brain activity patterns should be greater for within-pairs, relative to between-pairs, after the presentation of phonological similarity. If comprehenders predict the phonological information of the upcoming words, we would expect a greater similarity value to appear *before* the presentation of phonological overlap (i.e. before the onset of the third character), as greater phonological similarity in the third character should elicit greater neural similarity.

## Materials and methods

### Participants

Twenty-nine Mandarin Chinese native speakers residing in Beijing, participated in the experiment. All of participants had normal or correct-to-normal vision and no history of language disorders. Participants were provided with informed consent and were compensated approximately $20 USD for their participation. The study was approved by the Institutional Review Board of the Institute of Psychology, Chinese Academy of Sciences.

### Materials and design

**Fig. 1:**
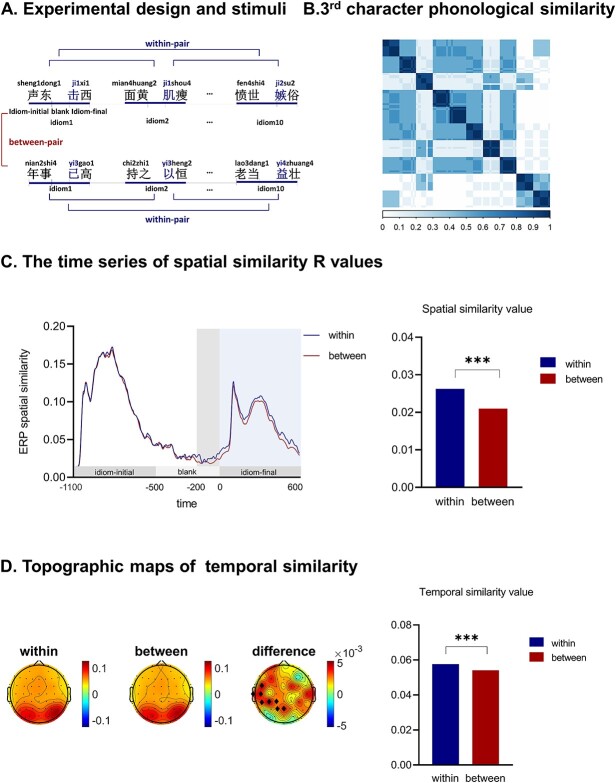
(A) Four-character Chinese idioms were presented. Idiom-initial part including two characters were presented first, followed by the idiom-final part with two characters. In the within-pair condition, idioms in a pair had an identical syllable in idiom-final part. In the between-pair condition, there was no phonological overlap among idioms. Numbers indicate tone for each syllable. (B) Model-based phonological similarity values of the third characters are shown in a 100 × 100 symmetric similarity matrix, with 10 characters sharing a syllable sound. The phonological similarity values of within-pairs (values around the diagonal line) were larger than those of between-pairs (values away from the diagonal line). (C) Spatial RSA results. It shows the time series of similarity values of within- (blue line) and between-pairs (red line), after the onset of idioms. The display of idiom-initial (−1,100 to −500 ms), the interval (−500 to 0 ms), and idiom-final (0–600 ms) are marked with gray boxes on the *x*-axis. Spatial RSA starts to show more similarity for within-pairs around 200 ms before the onset of the phonological overlap. The bar chart presents differences in averaged spatial similarity values from −200 to 0 ms among within-pairs (blue bar) and between-pairs (red bar). (D) Temporal RSA results. It shows topographic maps of temporal similarity. Both the within- and between-pair conditions demonstrate the greatest similarity value over the posterior area. The difference map shows greater temporal similarity for within-pairs than between-pairs over the central-posterior area. The electrodes where this difference was significant at the cluster level are marked with black diamonds. The bar chart presents differences of averaged similarity values in the significant cluster among within-pairs (blue bar) and between-pairs (red bar).

One hundred four-character Chinese idioms were selected, in which the first two characters formed idiom-initial and the last two characters formed idiom-final part. These one hundred idioms were divided into 10 sets with each set consisting of 10 idioms sharing the same sound form at the third character of idiom-final part (e.g. 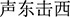 “sheng1dong1 **ji1**xi1” and 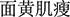 “mian4huang2**ji1**shou4,” see Fig. 1A for an example). Within each set, all possible pairs of idioms shared the same sound form at the idiom-final part (i.e. within-pairs), resulting in 45 within-pairs in each set and 450 within-pairs in total. The idioms were repaired with an idiom from a different set to form between-pairs (4,500), ensuring that the items did not share a sound form in the between-pairs. The idioms were pretested using a cloze probability test to verify that idiom-final of idioms was highly predictable. Twenty-one participants were presented the fragment up to idiom-final and were asked to complete each with the first word that came to mind. The cloze probability of the expected idiom-final parts was the proportion of participants who completed the idiom with the expected idiom-final parts (Taylor 1953). The average cloze probability for these idioms was 97%, indicating that the expected idiom-finals were highly predictable. The similarity values of cloze probability were measured by the absolute difference for each possible pair of idioms. Statistical analysis revealed that the similarity values of cloze probability were comparable between the within- and between-pairs (*t* < 1, *P* = 0.698).

A separate set of 20 idioms with the same structure was selected. Idiom-initial and idiom-final parts were recombined among these idioms to form 20 incongruent combinations. Participants were asked to judge whether the idiom-initial and idiom-final in a trial was an idiom or not. The experiment consisted of 120 trials, including 100 idioms and 20 incongruent combinations. Each participant was presented with two blocks of 60 trials consisting of the 50 idioms and 10 incongruent combinations, presented in a randomized order.

### Quantifying the phonological similarity among characters in idioms between within- and between-pairs

The focus of this study was to measure the neural activity elicited by the pre-activation of phonological information of the upcoming third character of an idiom, and to examine the similarity of neural activity between within- and between-pairs. Hence, the experimental hypothesis rested on the assumption that the third characters in within-pairs should be more phonologically similar to each other than those in between-pairs. To assess this assumption, we calculated phonological similarity values between all possible pairs of third characters for within- and between-pairs, using the Soundshape Code (Chen et al. 2018; Wang et al. 2020a). As presented in Fig. 1(B), the mean phonological similarity among the third characters was indeed significantly greater for within-pairs compared with between-pairs (third character: 0.93 vs. 0.26; *t* = 63.38, *P* < 0.001). Additionally, we aimed to ascertain that any differences in neural similarity were not due to phonological similarity among the initial two characters (i.e. the idiom’s initial parts). Statistical analysis demonstrated that phonological similarity for the other three characters were equivalent between within- and between-pairs (first character: 0.16 vs. 0.16; *t* < 1, *P* = 0.921; second character: 0.16 vs. 0.16; *t* < 1, *P* = 0.885; fourth character: 0.16 vs. 0.16; *t* < 1, *P* = 0.491).

### Quantifying the semantic similarity among characters in idioms and among the whole idioms between within- and between-pairs

The aim of the present study was to focus on the phonological similarity between characters in idioms. Stimuli were selected carefully to minimize semantic similarity between items within a pair, to ensure that the observed effect of greater neural similarity for within- vs. between-pairs reflects phonological similarity rather than semantic similarity. It was important to verify that semantic similarity among the four characters and the whole idioms were matched well for within- pair vs. between-pairs. To quantify semantic similarity, HowNet, an online database that provides calculations of inter-conceptual and inter-attribute relationships of Chinese lexicons (Dong et al. 2010), was used. Semantic similarity values for all possible pairs were measured via a path-based approach by Wu and Palmer (1994). Statistical analysis showed that there were no significant differences in the semantic similarity values among the characters and among the whole idioms for within- vs. between-pairs (first to fourth character: 0.45 vs. 0.43; 0.44 vs. 0.45; 0.41 vs. 0.41; 0.43 vs. 0.44; *t*s < 1.49, *p*s > 0.14; whole idiom: 0.28 vs. 0.28; *t* < 1). In addition, we collected semantic rating scores from a group of 15 native Chinese participants for all 450 pairs of idioms from within-pairs and for 450 pairs of idioms randomly selected from a full set of 4500 between-pairs (1 = “not related at all”, 7 = “closely related”). The mean semantic rating scores were 2.09 and 1.99 for within-pairs and between-pairs, respectively, and there was no difference between within- and between-pairs (*P* > 0.07). Thus, the stimuli were well matched semantically across within- and between-word pairs, and it is unlikely that semantic similarity contributed to the observed effect.

### Quantifying the orthographic similarity among characters in idioms between within- and between-pairs

To assess orthographic similarity between items for within- and between-pairs, we utilized Soundshape Code (Chen et al. 2018; Wang et al. 2020b). The results indicated that the orthographic similarity values among the idiom-initial characters showed no significant difference between within- and between-pairs (first character: 0.30 vs. 0.30; *t* < 1, *P* = 0.878; second character: 0.32 vs. 0.31; *t* = 1.45, *P* = 0.146). As introduced above, we manipulated phonological similarity among the third characters so that within-pairs had higher phonological similarity than between-pairs. Although care was taken to minimize orthographic similarity between phonologically related items during stimuli selection, the orthographic similarity among the third characters was slightly higher for within- than between-pairs (third character: 0.37 vs. 0.31; *t* = 6.35, *P* < 0.001). Orthographic similarity among the fourth characters was matched between within- and between-pairs (4th character: 0.28 vs. 0.29; *t* = −1.28, *P* = 0.200). Therefore, we controlled orthographic similarity of the third characters in data analysis (see details in Results section).

### Procedure

The experiment was conducted using E-Prime software. Participants were instructed that they will be presented with the idiom-initial with two characters and subsequently idiom-final with two characters on the computer screen. Participants were asked to judge whether idiom-initial and idiom-final in a trial is an idiom or not as quickly and accurately as possible, and press a designated keyboard button when the two parts cannot form an idiom. On each trial, participants saw a fixation cross (300 ms), a blank screen (500 ms), a display of idiom-initial (600 ms), a blank screen (500 ms), and a display of idiom-final (600 ms). An interval of 1,000 ms was inserted between trials. Participants received four practice trials before experimental trials. The experiment task lasted 20 minutes, and the entire experiment lasted approximately 60 minutes.

### E‌EG recordings and analysis

The EEG signals were recorded from 64 electrodes using Neuroscan software and collected using an elastic cap. The vertical and horizontal electrooculogram was captured via electrodes above and below the left eye and on the left and right external cantus, respectively. The left mastoid electrode was used as a reference, and all electrode impedances were kept below 5 kΩ. The signals were amplified and filtered with a bandpass filter between 0.05 and 70 Hz with a sampling rate of 1,000 Hz.

The EEG data were preprocessed using EEGLAB v2021.1 (Swartz Center for Computational Neuroscience: http://www.sccn.ucsd.edu/eeglab). The data were re-referenced to the average of both mastoids and down-sampled to 250 Hz. A high-pass cutoff point of 0.1 Hz and a low-pass cutoff point of 30 Hz were used to filter the data. An additional analysis without using offline high-pass filters produced identical pattern of results, which has demonstrated that high-pass filter settings in this range do not produce distortions in the similarity analysis (see also Wang et al. 2018; Hubbard and Federmeier 2021). Independent component analysis (ICA) using the infomax algorithm (Bell and Sejnowski 1995) was performed to remove eye blink and movement, muscle and bad channel components. Ocular components were determined when their topography showed large activity over the vertical or horizontal eye electrodes, and when they showed opposite polarity below or around the eyes. Muscle components were determined when their topography was very focal, encompassing a local group of electrodes on the edge of the cap, with high power at high frequencies (>20 Hz). Bad channel components have a focal topography restricted to the bad channel (Chaumon et al. 2015). On average, 10.6% ICs (6.5 out of 62 components) were removed from each participant’s dataset. Epochs containing amplitudes exceeding ±120 μV were rejected, which accounted for approximately 2.9% of all epochs. The remaining epochs were, on average, 117 trials per participant. The EEG was segmented into 2,000-ms epochs that included a 300- ms pre-stimulus display as a baseline, a 600-ms idiom-initial display, 500-ms interval, and 600-ms idiom-final display. Epochs were baseline corrected and the averaged signal in a baseline period was subtracted from the remaining ERPs.

### Spatial RSA

For RSA analyses, trials with incongruent combinations were excluded and only trials with idioms were included for RSA analyses, to rule out the influence of incongruent combinations to neural activity similarity. Spatial RSA is focused on the similarity of neural activity across the scalp at each timepoint, allowing to answer the time window of the similarity effect. An item-specific EEG vector for each item was computed across all channels (62 electrodes), which represents the pattern of neural activity. For each individual participant, we quantified the degree of similarity of neural activity patterns between all possible pairs for both within- (i.e. 45 within-pairs for 10 idioms in each set, and thus 450 within-pairs in total), and between-pairs (i.e. 4,500 between-pairs in total), by calculating Pearson’s *r*-value between the patterns of neural activity of items. Pairwise correlation *r*-values were averaged to yield mean similarity values for within- and between-pairs at each time point for each participant. To visualize any differences between the two conditions, we averaged these similarity values across all participants at each consecutive time point for within- and between-pairs. This generated a grand-average similarity over time for each condition (Fig. 1C).

Several recent studies have reported the elicitation of a prediction potential, appearing within tens to a few hundred milliseconds before predictable stimulus onset (Grisoni et al. 2017, 2019; León-Cabrera et al. 2019. On the basis of these findings, to investigate prestimulus predictive brain activity, the present study focused on the last 200 ms before idiom-final onset (−200 to 0 ms). The statistical significance of the neural activity similarity within- vs. between-pairs was tested by computing the average of mean neural similarity values obtained for each condition, and subjecting it to a t-test. A similarity analysis was also conducted for the time window after the presentation of the idiom-final part (0–600 ms) to confirm the similarity effect after the presentation of phonological similarity, as assumed.

Second, a cluster-based permutation test (Maris and Oostenveld 2007) was conducted to test for differences in similarity between within- and between-pairs. The cluster permutation test was run on the entire epoch before idiom-final presentation (−1100–0 ms). This involved performing t-tests at each time point between conditions, grouping adjacent timepoints where the t-test was significant (*P* < 0.05) to identify clusters, and determining the magnitude of each observed cluster by summing the *t*-values within the cluster. Condition labels were randomly shuffled for each participant, and the procedure described above was repeated for 1,000 times to form the H0 distribution of cluster statistics. Significant observation was considered when the observed summed t value falls outside the 95% range of the distribution. A separate cluster-permutation test was performed on the epoch after idiom-final presentation (0–600 ms) to examine the similarity effect after phonological similarity presentation.

### Temporal RSA

Temporal RSA focuses on calculating the similarity of two time series at each electrode across the scalp, and identifying brain regions where the temporal pattern of neural activity is more similar for within-pairs compared with between-pairs. Specifically, for each participant, at each electrode for each trial, the EEG time series in the −1,100- to 0–ms interval was used as the temporal pattern of neural activity. For each electrode, the temporal similarity values were obtained by correlating the time series between trials within each pair, and then averaging the similarity values across all within-pair trials at each electrode. Grand-average temporal similarity was obtained by averaging similarity values across participants. Grand average similarity values across all the electrodes create a grand average topographic map of similarity (see Fig. 1D). The procedure was repeated for the between-pair condition.

The within- vs. between-pair temporal similarity values were compared using a cluster-based permutation approach to control for multiple comparisons over electrodes. At each electrode, we compared the average temporal similarity values and computed the mean difference between the within- vs. between-pair conditions. Subsequently, a permutation test was conducted by randomly shuffling the data between the two conditions for 1,000 iterations, with mean differences calculated for each permutation. The *P*-value for the observed mean difference was then determined by comparing it to the distribution of mean differences obtained from the permutation test. Electrodes with *P*-values less than or equal to 0.05 were considered statistically significant. Adjacent electrodes were considered neighbors, and significant neighbors compose clusters. To assess the significance of a cluster, we performed 1,000 permutation tests following the same procedure and generated the size of the largest clusters. A *P*-value for a cluster was obtained by comparing the size of the original cluster with the distribution of cluster sizes.

## Results

### Behavioral results

Participants were asked to respond to incongruent combinations, thus behavioral data were only available for incongruent trials. The mean response latency was 663 ms (SD = 195 ms) and the correct rate was 92.6%.

### Spatial RSA results

As shown in Fig. 1(C), as expected, during the idiom-initial display, there was no significant difference in neural similarity between items for within- and between-pairs. During the idiom-final display, the patterns of brain activity between items were more similar for within-pairs than between-pairs. Critically, the greater neural similarity for the within-pair condition already showed up before the onset of idiom-final part, from 200 ms before the onset of phonological similarity. Statistical analyses confirmed that the greater neural similarity for within-pairs was observed before the presentation of idiom-final parts, relative to neural similarity for between-pairs [−200 to 0 ms; *P* = 0.005], reflecting the prediction of phonological information of upcoming idiom-final parts. Moreover, the patterns of brain activity between items were more similar for within-pairs than between-pairs after the presentation of phonological similarity (i.e. idiom-final) from the onset of idiom-final to 600 ms (0–600 ms; *P* < 0.001), elicited by the processing of phonological similarity.

The difference in neural similarity prior to the idiom-final is unlikely to be attributed to the disparity in the number of within-pair and between-pair correlations used to compute the mean temporal correlation values, as the number of trials per condition can affect the variance of the estimated mean value, but not the value of the estimated mean itself. Consequently, the different number of within- and between-pairs should not influence statistical inference at the participant level (Thomas et al. 2004; Groppe et al. 2011). To further validate this conclusion, we conducted an additional analysis using a randomly selected subset of between-pair correlations that matched the number of within-pair correlations. This analysis confirmed that in the critical predictive time window [−200 to 0 ms], the within-pair correlation values remained significantly greater than the between-pair correlations (*t* = 3.84, *P* = 0.0003)

To test for the possibility that other variables such as semantic similarity or/and orthographic similarity between the third characters would explain the spatial RSA effects, we used linear mixed-effects models to predict the neural similarity value in the predictive time window with phonological similarity, semantic similarity, and orthographic similarity of the third characters on a single-trial level. For each trial, the average neural similarity derived from −200 to 0 ms was extracted. The model included fixed factors and random intercepts for participants, and participant random slopes for phonological similarity. The mixed-effect model analysis was performed using the “‘lme4”’ package as implemented in R. Significance of fixed effects of the model was with the *anova()* function from package *lmerTest*. Phonological similarity of the third characters remained a significant predictor of neural similarity (*F* = 6.55, *Pp* = 0.011), even with semantic similarity and orthographic similarity included. Semantic similarity failed to reach significance (*F* < 1), and orthographic similarity reached significance (*F* = 4.58; *Pp* = 0.032). Thus, phonological similarity is a significant variable for the RSA effect, even with other variables controlled.

The cluster-based permutation test across the entire time series before the onset of phonological similarity (−1,100 to 0 ms) confirmed the presence of a significant similarity effect. The results revealed two significant clusters, one from −140 to −116 ms (*P* = 0.008) and another from −48 to −12 ms (*P* = 0.007). It should be noted that the cluster-based permutation tests are known to be conservative and may underestimate the true effect size (Maris and Oostenveld 2007). Additionally, a cluster-based permutation test was performed after the presentation of phonological similarity (0–600 ms) to examine the difference in neural similarity between within- and between-pair conditions. As anticipated, significant clusters were identified (*p*s < 0.05) confirming the expected results.

### Temporal RSA results

To identify brain regions where the temporal pattern of neural activity is more similar for within-pairs compared with between-pairs, we conducted temporal RSA. As shown in Fig. 1(D), both the within- and between-pair conditions demonstrate the greatest similarity values over posterior regions. When comparing the within- and between-pair temporal similarity values, the results of the cluster-based permutation test revealed a significant cluster over left middle-posterior regions, with the temporal pattern of neural activity more similar for within-pairs than between-pairs (*P* = 0.001).

## Discussion

In the present study, we aimed to investigate whether upcoming phonological information is predicted in reading four-character Chinese idioms using ERP RSA. We presented the first two characters of the idiom, followed by the last two characters. Phonological overlap was manipulated among idioms at the third character so that pairs of idioms share the same sound of the third character (i.e. within-pairs) or not (between-pairs). As expected, we found greater similarity among patterns of neural activity for within-pairs than between-pairs. Critically, the similarity effect appeared ~ 200 ms before the presentation of phonological similarity and reflected the prediction of phonological information of the upcoming content.

### The RSA effect after the presentation of phonological similarity

The critical assumption of RSA is that similarity between items elicits similarity in patterns of brain activity. On the basis of this assumption, phonological similarity between idioms should generate greater similarity in brain activity. It is important to confirm this assumption, before discussing neural similarity effects appearing before the presentation of phonological similarity, i.e. RSA predictive effects. Previous studies have mainly investigated how semantic similarity affects neural similarity, and revealed that semantic similarity indeed elicits greater neural similarity (e.g. Devereux et al. 2013; Wang et al. 2020a). In contrast, little work has examined how phonological similarity between items affects neural similarity. In the present study, our results confirmed this assumption by demonstrating that within-pairs, which shared phonological information, generated greater similarity in patterns of brain activity, compared with between-pairs.

### The RSA effect before the presentation of phonological similarity

While it is well established that comprehenders predict upcoming words during language comprehension, the question whether comprehenders can predict at various linguistic levels or only at specific levels of representation remains unclear. Can comprehenders predict phonological information of upcoming content? As reviewed in Introduction, previous investigations on phonological prediction have painted a rather mixed picture, with some positive findings contrasting with some null findings. In this study, we demonstrated that neural similarity effects started to appear at ~ 200 ms before phonological similarity among idioms become available, reflecting the pre-activation of phonological information. But could the neural similarity effect reported here have resulted not from phonological similarity, but rather from other potential confounding factors such as semantic similarity of idioms which is able to elicit neural similarity effects? We have matched the degree of semantic similarity between within- and between-pair conditions, and the result is therefore unlikely to have reflected differences in semantic similarity among idioms. Furthermore, our results indicate that the RSA effect was not observed during the presentation of the idiom-initial part. Rather, the effect emerged only after the idiom-initial part had been presented, which suggests that it was not evoked by the processing of the idiom-initial part.

Concerning the time course, the present study revealed evidence of phonological prediction just prior to the presentation of predictable words. This finding is in line with previous studies utilizing ERP measures, which have demonstrated the emergence of a prediction potential in response to spoken and written sentence fragments with predictable endings. This potential is typically observed within the last few hundred milliseconds before the onset of the predicted stimulus (Grisoni et al. 2017, 2019; León-Cabrera et al. 2019). Additionally, a recent EEG RSA investigation by Hubbard and Federmeier (2021) showed that predictive evidence was only present for words immediately preceding predictable words, rather than preceding words. Collectively, these results suggest that (word-form) predictions are generated in close temporal proximity to the anticipated presentation of critical words.

Temporal RSA revealed the greatest similarity value over posterior brain region and this observation is in line with recent studies that conducted temporal RSA to linguistic prediction and reported peak temporal similarity in occipital regions (Wang et al. 2018; Hubbard and Federmeier 2021). Additionally, temporal RSA revealed greater similarity of temporal pattern for within-pairs than between-pairs over left middle-posterior regions. Due to limited spatial resolution of EEG technique, it is difficult to localize the source of this effect. But the current finding about the left middle-posterior region of the similarity effect is in line with MEG results that demonstrated the prediction of particular word form features (Dikker and Pylkkänen 2013). In that study, MEG source localization of activity prior to predicted target words revealed left medial temporal and occipital sources, with temporal activity slightly preceding occipital activity, which suggest that language comprehenders may pre-activate lexical information in temporal context, followed by prediction of particular word form in sensory cortex.

The finding that comprehenders can predict phonological information in language comprehension should not be overinterpreted to suggest that phonological prediction is ubiquitous during language comprehension. In this respect, we broadly agree with a dynamic view of linguistic prediction that comprehenders can vary in their specificity of prediction in a dynamic fashion, depending on the constraining of context, processing resources available, etc. Phonological prediction is less likely (if not unlikely) to occur unless context is sufficiently constraining, whereas the language processing system could make more detailed predictions that specify precise phonological representations when context provides enough constraint, such as in comprehending idioms which are highly structured and widely used in daily lives. The scope of our findings is that it demonstrates that the language processing system can perform phonological prediction when context provides enough constraint.

In conclusion, this investigation yields neural evidence substantiating the occurrence of linguistic prediction at the level of phonological forms during language comprehension, thereby emphasizing the presence of phonological prediction in language processing, particularly under circumstances that encourage high-certainty predictive processing.

## Authors’ contributions

Q.Q.Q. and Z.R.H. designed the study. W.W. and C.F. performed data analyses. Q.Q.Q. and W.W. wrote the paper.

## Funding

This work was supported by the National Natural Science Foundation of China (Nos 32171058 and 62061136001), Youth Innovation Promotion Association (Chinese Academy of Sciences), Youth Elite Scientist Sponsorship Program (No. YESS20200138, China Association for Science and Technology), Beijing Nova Program (20220484081), and the Scientific Foundation of Institute of Psychology (No. E2CX3625CX, Chinese Academy of Sciences to Q.Q.).

*Conflict of interest statement:* None declared.

## Data availability

For protection of participants’ privacy, the data are available upon request to the authors.

## CRediT author statement

Wei Wei (Formal analysis, Investigation, Methodology, Project administration, Writing—original draft, Writing—review and editing), Zirui Huang (Conceptualization, Investigation, Methodology, Project administration), Chen Feng (Formal analysis, Methodology, Software), Qingqing Qu (Conceptualization, Formal analysis, Funding acquisition, Investigation, Methodology, Project administration, Software, Supervision, Validation, Writing—original draft, Writing—review and editing).
